# Hybrid operating room with ceiling mounted imaging system assisted pre-operative and intra-operative lung nodule localization for thoracoscopic resections: a 5-year case series

**DOI:** 10.1186/s13019-024-02564-7

**Published:** 2024-02-10

**Authors:** Audrey Qi Xin Chia, Apoorva Gogna, Angela Maria Takano Pena, Vishnu Vemula Sri Sai, Sivanathan Chandramohan, Shaun Ju Min Xavier Chan, Boon-Hean Ong

**Affiliations:** 1 Department of Vascular and Interventional Radiology, Singapore General Hospital, Singapore, Singapore; 2 Department of Anatomical Pathology, Singapore General Hospital, Singapore, Singapore; 3 Lee Kong Chian School of Medicine, Nanyang Technological University, Singapore, Singapore; 4 Department of Cardiothoracic Surgery, National Heart Centre Singapore, 5 Hospital Drive, Singapore, 169609 Singapore

**Keywords:** VATS, Sublobar resection, Nodule localization, Cone-beam computed tomography, Hybrid operating room

## Abstract

**Background:**

Video-assisted thoracoscopic (VATS) lung resections are increasingly popular and localization techniques are necessary to aid resection. We describe our experience with hybrid operating room (OR) cone-beam computed tomography (CT) assisted pre-operative and intra-operative lesion localization of lung nodules for VATS wedge resections, including our novel workflow using the hybrid OR cone-beam CT to re-evaluate patients who have undergone pre-operative localization for those who are unsuitable for intra-operative localization.

**Methods:**

Retrospective analysis of all consecutive patients with small (≤ 20 mm), deep (≥ 10 mm distance from pleura) and/or predominantly ground-glass nodules selected for lesion localization in the Interventional Radiology suite followed by re-evaluation with cone-beam CT in the hybrid OR (pre-operative), or in the hybrid OR alone (intra-operative), prior to intentional VATS wedge performed by a single surgeon at our centre from January 2017 to December 2021.

**Results:**

30 patients with 36 nodules underwent localization. All nodules were successfully resected with a VATS wedge resection, although 10% of localizations had hookwire or coil dislodgement. The median effective radiation dose in the pre-operative group was 10.4 mSV including a median additional radiation exposure of 0.9 mSV in the hybrid OR for reconfirmation of hookwire or coil position prior to surgery (*p* = 0.87). The median effective radiation dose in the intra-operative group was 3.2 mSV with a higher mean rank than the intra-operative group, suggesting a higher radiation dose (*p* = 0.01).

**Conclusions:**

We demonstrate that our multidisciplinary approach utilizing the hybrid OR is safe and effective. Intra-operative localization is associated with lower radiation doses. Routine use of cone-beam CT to confirm the position of the physical marker prior to surgery in the hybrid OR helps mitigate consequences of localization failure with only a modest increase in radiation exposure.

## Introduction

### Background

Low-dose screening chest CT has been shown to decrease lung cancer-related mortality [1] and its use is hence increasing [2]. Concomitantly, there is a growing need for safe and effective histological confirmation of indeterminate lung lesions [1]. Percutaneous transthoracic CT-guided needle biopsy is often used, but certain lesions may do better with upfront sublobar lung resections, which would simultaneously provide diagnosis and treatment. In particular, a wedge resection with sufficient resection margins for predominantly ground glass lesions has been shown to provide excellent long-term outcomes [3, 4].

Video-assisted thoracoscopic (VATS) lung resections are increasingly popular compared to open resections, given the significantly reduced peri-operative morbidity [5]. However, small, deep or predominantly ground-glass intra-parenchymal lesions are difficult to localize during VATS, as these lesions are often neither palpable nor visible on the pleural surface. Up to 63% of patients undergoing VATS have required conversion to thoracotomy for lesions < 10 mm in size or > 5 mm from the pleural surface [6]. Hence, localization techniques have become necessary for accurate VATS wedge resections for these lesions.

### Rationale and knowledge gap

Pre-operative transthoracic CT-guided lesion localization has been previously well described using hookwires, microcoils, contrast dye or radiotracer prior to targeted surgical resection [7–15]. However, there is a risk of localization failure from hookwire or coil dislodgement or dye diffusion. In addition, patient anxiety and discomfort are increased due to the waiting time between the localization and the subsequent transfer to the operating room (OR) for surgical resection [16]. In recent years, hybrid ORs have allowed for intra-operative localization, where both the surgical resection and localization procedure can be performed at the same setting, to overcome the limitations associated with pre-operative localization [17–20]. However, despite the multiple techniques described, there is still no clear approach as to which technique is superior.

### Objective

Our study aims to describe our institution’s 5-year experience with hybrid OR cone-beam CT assisted pre-operative and intra-operative lesion localization of lung nodules for VATS wedge resections. We describe and compare two main methods:

1. For pre-operative localization, patients undergo lung nodule localization in the Interventional Radiology department using conventional CT, after which the patient is transferred to our hybrid OR with a ceiling mounted imaging system where a repeat cone-beam CT is done after the patient is anaesthetised and placed in a lateral position to re-evaluate the location of the physical marker (eg. hookwire) in relation to the lung nodule of interest to guide accurate VATS wedge resection. This is a novel technique which we propose can overcome limitations traditionally associated with pre-operative localization by allowing re-evaluation of the location of the physical marker in relation to the lesion of interest to mitigate the consequences of localization failure in the event of dislodgement of the physical marker. In particular, we believe this may be well suited to many practices which have hybrid OR but wish to take on challenging lesions that are hard to localize on cone-beam CT with intra-operative localization.
2. For intra-operative localization, patients undergo localization in the hybrid OR with cone-beam CT guidance after the patient is anaesthetised and placed in lateral position, similar to what as been described previously by other groups [17–20].

## Methods

### Participants

This is a retrospective analysis of all 30 consecutive patients with indeterminate pulmonary nodules (n = 36) requiring lesion localization prior to intentional VATS wedge resection, performed by a single surgeon at our centre over a 5-year period between January 2017 and December 2021.

Patients with indeterminate pulmonary nodules at our centre were discussed at a multi-disciplinary tumour board to assess for suitability for nodule localization with intentional VATS wedge resection for both diagnostic and therapeutic purposes. Inclusion criteria consisted of one or more of the following characteristics: small nodule size (≤ 20 mm), deep nodule location (≥ 10 mm distance from pleura) or radiologic appearance being predominantly ground-glass. These are lesions which potentially would be difficult to identify by visual or tactile assessment intra-operatively by the surgeon for accurate VATS wedge resection.

**Fig. 1:**
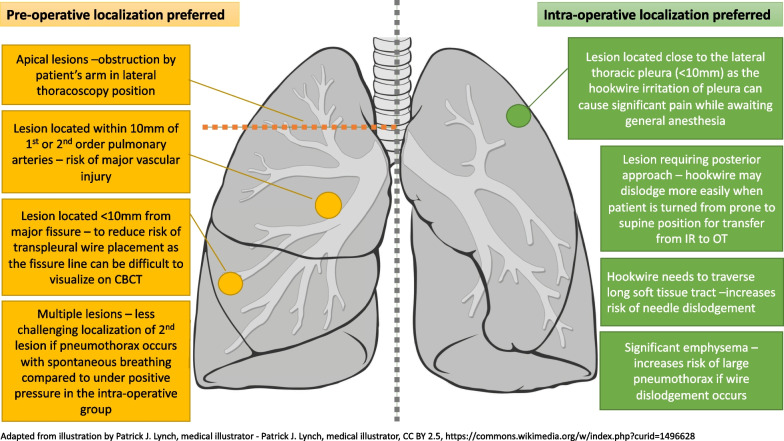
Diagrammatic representation of patient and lesion characteristics favoring either pre-operative or intra-operative localization. Adapted from the illustration—Lungs diagram simple.svg by Patrick J. Lynch, medical illustrator and C. Carl Jaffe, MD, cardiologist under creative commons license CC BY 2.5, https://commons.wikimedia.org/w/index.php?curid=1496628

All else being equal, we favour intra-operative localization over pre-operative localization whenever possible as it is more comfortable for the patient as it is done while the patient is under general anaesthesia. However, the choice of pre-operative or intra-operative localization was predominantly based on a number of factors (Fig. 1).

**Fig. 2:**
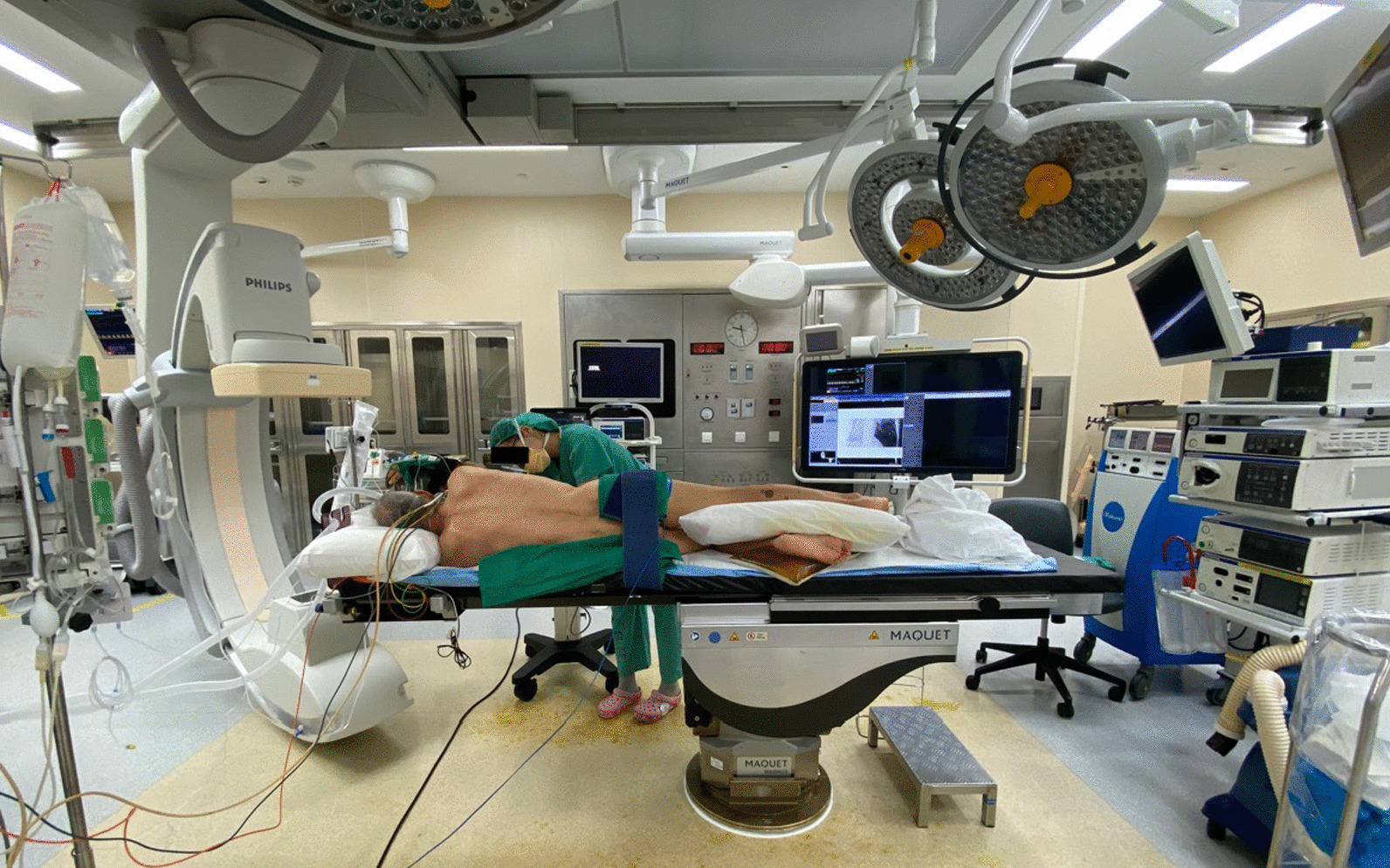
Hybrid operating room equipped with ceiling mounted Allura FlexMove fluoroscopy unit showing restricted movement of the c-arm due to patient positioning with arm in the lateral position
- Lesions located within 10 mm of 1st or 2nd order pulmonary arteries, as the higher CT resolution in the IR suite is required for better visualization of the vessels, to facilitate precise needle manipulation to avoid major vascular injury (Fig. 3A and B).

Pre-operative localization in the interventional radiology (IR) suite was preferred in the following situations:

- Apical lesions, as assessment and localization approaches of these lesions on intra-operative cone-beam CT are limited by restricted movement of our hybrid OR’s ceiling mounted c-arm, due to required surgical positioning of the patient with arm in the lateral position (Fig. 2).

**Fig. 3:**
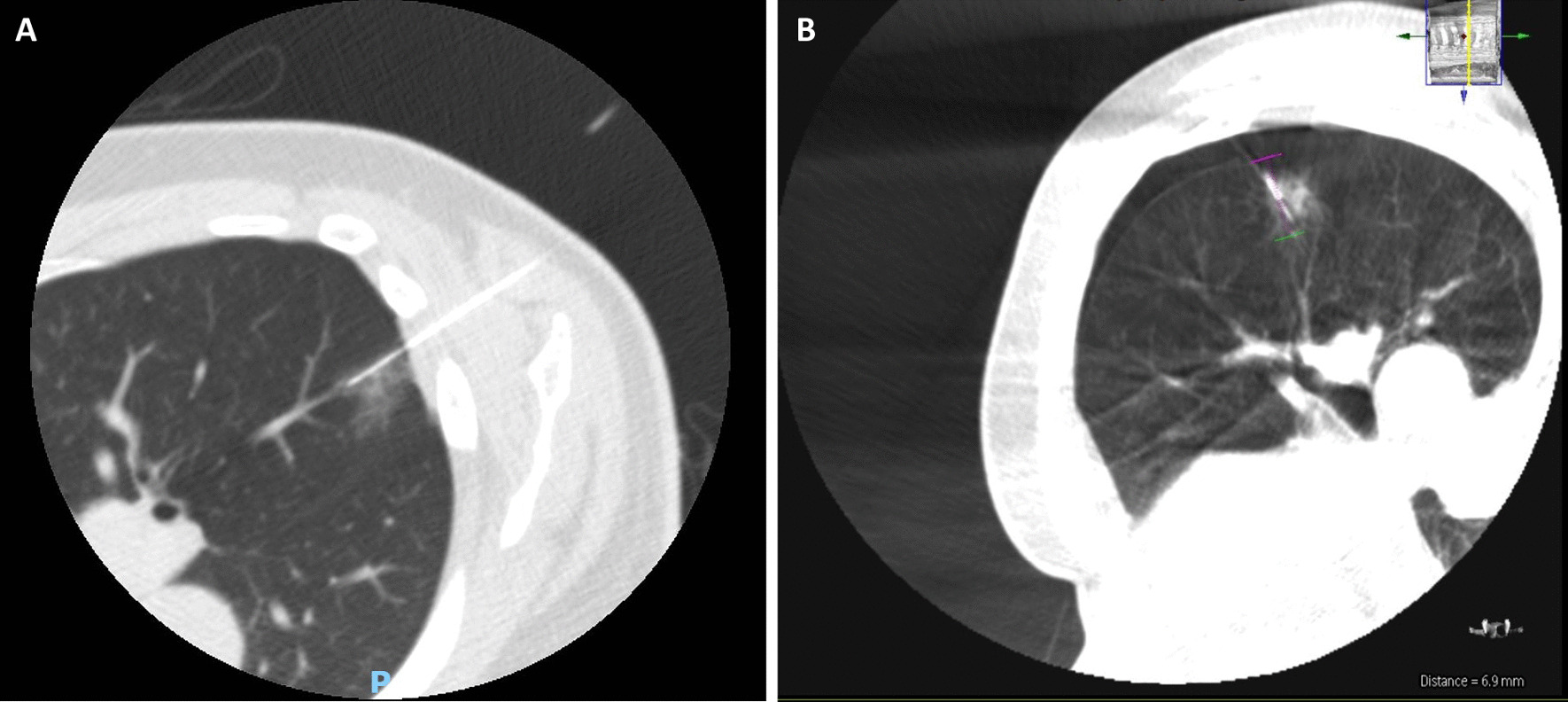
IR suite CT and intra-operative CBCT images depicting difference in resolution and visualization of pulmonary arteries. **A** IR suite CT with hookwire in-situ after localization of a lesion closely related to pulmonary arteries. **B** Intra-operative confirmatory CBCT of the same patient. Note visualization of adjacent pulmonary arteries is more challenging due to lower resolution
- Lesions located close to the major fissure (< 10 mm), as the higher CT resolution in the IR suite is required for better visualization of the fissure, to facilitate precise needle manipulation and reduce the risk of transpleural wire placement.
- Multiple lesions, as localization of the second lesion is less difficult if a pneumothorax occurs after localization of the first lesion while the patient is spontaneously breathing. On the contrary, a greater degree of positional change to the second lesion occurs in patients who develop a pneumothorax after localization of the first lesion while the patient is under positive pressure ventilation with intra-operative localization, which tends to significantly increase the size of the pneumothorax.

On the other hand, intra-operative localization was preferred in the following situations:

- a) Lesions located close to the lateral thoracic pleura (< 10 mm), as the hookwire irritation of the pleura can cause significant pain post-localization while awaiting general anaesthesia for surgery.
- b) Lesions requiring a posterior approach, as the hookwire is more easily dislodged when the patient is turned from prone to supine position for transfer from the IR suite to the OR.
- c) Lesions where the hookwire needs to traverse a long soft tissue tract, which also increases the risk of hookwire dislodgement during transfer from the IR suite to the OR.
- d) Patients with significant emphysema, which increases the risk of large pneumothorax if hookwire dislodgement occurs.

### Localization procedure

All lesions were localized with a metallic device as a physical marker, usually a Kopans hookwire (IZI Medical, Maryland, USA) which was used for 33 lesions. In three lesions when the hookwire was unavailable, a 0.018″ 3 × 33 mm Vortx Platinum Coil (Boston Scientific, Massachusetts, USA) was used instead.

**Fig. 4:**
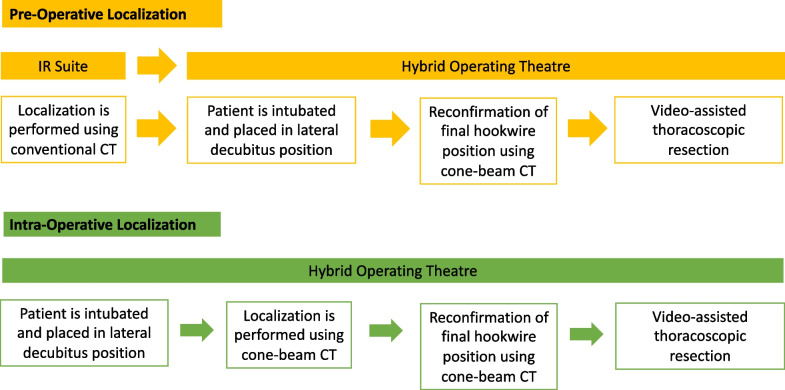
Workflow for pre-operative and intra-operative localization

From 2019 onwards, when our institution acquired a camera system capable of near-infrared fluorescence imaging (Karl Storz, Tuttlingen, Germany), an adjunct localization technique of either indocyanine green (ICG) (Diagnostic Green GmbH, Aschheim-Dornach, Germany) (0.2 ml of 5 mg/ml ICG solution) or Lipiodol (Guerbet, Villepinte, France) + ICG mixture (2.4 ml of Lipiodol to 0.3 ml of ICG) was used in addition to the metallic device. All three of the localizations performed using a coil involved the use of ICG. Several hookwire localizations were performed without adjuncts (n = 9). Adjuncts were utilised in 24 lesions including ICG only (n = 21) or a mixture of Lipiodol and ICG (n = 3). Figure 4 is a diagrammatic representation of the difference in workflow between pre-operative and intra-operative lesion localization.

Pre-operative lesion localization was performed in the IR suite on the same day just prior to surgery. The IR suite is equipped with a multi-detector CT scanner with CT-fluoroscopy (Canon Medical Systems Corp, Tochigi, Japan). The needle trajectory was selected after a review of the lesion location relative to pulmonary anatomy and a discussion with the surgeon on the planned operative approach.

Under sterile conditions, the 21G needle was advanced 0.5 cm beyond the distal edge of the lesion and position confirmed. After an ICG compatible camera system was available, an adjunct staining measure (ICG or a Lipoidol + ICG mixture) was used to increase visibility or as a precaution in the event of hookwire dislodgement. To limit over injection of the dye, we use the following technique: 0.3 ml of ICG is prepared in an extension tubing with one end open to the air. This allows the breathing force of the patient to draw the column of dye into the lesion gently rather than using manual forceful injection of the dye. Then, the hookwire was advanced up to the needle tip, and the needle was withdrawn while the hookwire was held in place. To mitigate the risk of hookwire dislodgement due to patient movement, an additional slack of 1-2 cm in the hookwire from puncture point is provided to allow for movement. Hookwires were also placed slightly deeper than absolutely necessary. The patient was then transferred directly to the hybrid OR. Reconfirmation of hookwire position was performed using cone-beam CT after the patient was placed under general anaesthesia and appropriately positioned just prior to surgical resection.

Intra-operative localization was performed in a hybrid OR equipped with a ceiling mounted Allura FlexMove fluoroscopy unit (Koninklijke Philips N V, Amsterdam, Netherlands). Following general anaesthesia with mechanical ventilation, the patient was similarly appropriately positioned. Cone beam CT images (Philips XperCT) of the region of interest were obtained using an 8 s abdominal roll protocol in suspended end inspiration. Using the needle guidance software (Philips XperGuide), the needle trajectory was planned with similar considerations as pre-operative localization. The skin entry point and lesion target point were marked on the initial XperCT images, which populated a planned needle trajectory. Two fluoroscopy positions are used for needle guidance: the entry-point view, which gives a bulls-eye view of the needle on target, and an orthogonal progress view, which allows tracking along the long-axis of the needle to the target.

Subsequently the needle was advanced under sterile conditions using the above trajectory, again in held end-inspiration. Once in position, XperCT was repeated to confirm needle tip position. Once acceptable position was obtained, ICG or a lipiodol + ICG mixture was injected into the lesion, hookwire placed and needle removed. A final cone-beam CT to reconfirm hookwire position in relation to the lesion was performed just prior to surgical resection.

### Surgical procedure

After final imaging reconfirmation of hookwire position, the surgical field including the hookwire was cleaned and draped. Standard three port VATS incisions were made, the ipsilateral lung was isolated and a 10 mm, 30-degree video telescope was introduced to inspect the thoracic cavity thoracoscopically. Visualization of the hookwire and area highlighted by ICG dye using near-infrared fluorescence thoracoscopy (when available) allowed for identification of the area of interest containing the lesion for resection. A wedge resection of this area was then performed using serial firing of an endoscopic stapling device (Echelon, Ethicon Inc, Cincinnati, OH, USA and EndoGIA, Medtronic Inc, Minneapolis, MN, USA). This was typically followed by a systematic lymph node sampling. In five patients, a concomitant lobectomy was also performed for a separate lesion in another lobe after the wedge resection – in these patients a systematic lymph node dissection was performed instead. Haemostasis and examination for air leak were performed as per routine prior to chest tube placement at the conclusion of the operation.

Post resection, x-rays of the specimen was performed to confirm that the entire hookwire had been removed together with the specimen. After histopathological results of the resection were finalized, patients diagnosed with primary lung cancer would undergo a positron emission tomography scan and magnetic resonance imaging of the brain to complete their staging work-up if not performed prior to surgery. Patients diagnosed with primary lung cancer with adequate lung function were offered completion lobectomy if high risk features, such as spread through air spaces, were present on histopathology.

### Variables and statistical analysis

Outcome measures for this study comprise localization and surgical outcomes.

Localization outcomes included the time taken for localization, effective radiation dose, and procedural complications. Effective radiation dose calculation for the pre-operative group included radiation exposure in the IR suite for localization (dose-length product, DLP in mGycm), as well as radiation exposure from cone-beam CT in the hybrid OR for reconfirmation of hookwire position (dose area product, DAP in Gycm^2^). Dose calculation for the intra-operative group included only exposure in the hybrid OR (DAP in Gycm^2^). All units were converted to milliSieverts (mSV) utilising a factor of 0.20 mSv/Gycm^2^ in the conversion of DAP to mSV (exposure for thorax region) and utilising a dose converter for DLP to mSV [21]. Procedural complications included those that occurred during the localization procedure itself, as well as in the interim period before resection in the pre-operative group.

Surgical outcomes included successful wedge resections of the lesion of interest, any need for conversion to thoracotomy, duration of surgery, intra-operative blood loss, post-operative hospital stay, post-operative complications and mortality. Successful wedge resection was defined as identification of the lesion of interest within the lung parenchymal tissue resected by VATS wedge resection without need for conversion to thoracotomy or a more extensive resection.

The histology of resected nodules was also reviewed.

Data are presented as mean with standard deviation for continuous variables and frequency with percentage for categorical variables unless otherwise indicated. Comparisons between the pre- and intra-operative groups were performed using 2-tailed independent samples t-test for continuous variables following a normal distribution and Mann Whitney U test for those not following a normal distribution. Chi-Square or Fisher’s Exact Test (when a cell value was lower than 5) was used for categorical variables. All analyses were conducted using statistical software IBM SPSS Statistics for Macintosh, Version 28.0. Armonk, NY: IBM Corp.

## Results

### Patient and nodule characteristics

From January 2017 to December 2021, a total of 30 patients (15 male, 15 female; mean age = 62.4, SD 10.73) underwent pre-operative or intra-operative localization followed by VATS wedge resection. Four patients (13.3%) had a history of ipsilateral lobectomy while 18 patients (60.0%) had a history of previous malignancy. The mean Charlson Comorbidity Index for these patients was 5.17 (SD 2.5). There were significantly more upper lobe lesions in the pre-operative localization group, as this was part of our selection criteria for deciding on modality of localization. Otherwise, there were no significant differences in the baseline characteristics between the two groups. No confounders were identified.

Data from a total of 30 patients were reviewed with four patients having multiple nodules for localization, with a total of 36 nodules localized. The majority of nodules were located in the right lower lobe (n = 15, 42.0%) with others in the right upper lobe (n = 5, 14.0%), right middle lobe (n = 2, 6.0%) left upper lobe (n = 7, 19.0%), and left lower lobe (n = 7, 19.0%). Nodule size ranged from 3 to 19 mm (mean = 10.5, SD 4.55 mm) with distance from pleura ranging from 0 (abutting the pleura) to 22 mm (mean 7.56, SD 6.53 mm). Nodule radiological characteristics included ground-glass (n = 13, 36.0%), solid (n = 12, 36.0%) and mixed (n = 10, 28.0%).

Table 1 summarizes the patient and nodule characteristics.

**Table 1:** Patient and nodule characteristics

Patient characteristics				
Variable		Total (n = 30) *n (%) or mean (SD)*	Pre-Op Localization (n = 13) *n (%) or mean (SD)*	Intra-op Localization (n = 17) *n (%) or mean (SD)*
Age (years)		62.4 (10.7)	60.4 (13.6)	64.0 (8.0)
Gender	Male	15 (50%)	6 (46.2%)	9 (52.9%)
	Female	15 (50%)	7 (53.8%)	8 (47.1%)
Smoking status	Current	5 (16.7%)	1 (7.7%)	4 (23.5%)
	Previous	6 (20.0%)	4 (30.8%)	2 (11.8%)
	Life-long non-smoker	19 (63.3%)	8 (61.5%)	11 (64.7%)
Previous ipsilateral Lobectomy		4 (13.3%)	2 (11.8%)	2 (11.8%)
Previous malignancy		18 (60.0%)	10 (76.9%)	8 (61.5%)
Charlson comorbidity Index		5.2 (2.5)	5.3 (2.9)	5.1 (2.1)

Nodule characteristics				
Variable		Total (n = 36) n (%) or mean (SD)	Pre-Op Localization (n = 16) n (%) or mean (SD)	Intra-op Localization (n = 20) n (%) or mean (SD)
Nodule size (mm)		10.5 (4.6)	10.4 (5.0)	10.6 (4.3)
Distance to pleura (mm)		7.6 (6.5)	7.4 (7.1)	7.7 (6.2)
Location^†^	RUL	5 (14.0%)	4 (25.0%)	1 (5.0%)
	RML	2 (6.0%)	0 (0.0%)	2 (10.0%)
	RLL	15 (42.0%)	3 (19.0%)	12 (60.0%)
	LUL	7 (19.0%)	5 (31.0%)	2 (10.0%)
	LLL	7 (19.0%)	4 (25.0%)	3 (15.0%)
Radiological characteristic	Ground-glass	13 (36.0%)	4 (25.0%)	9 (45.0%)
	Mixed solid-ground glass	10 (28.0%)	7 (44.0%)	3 (15.0%)
	Solid	13 (36.0%)	5 (31.0%)	8 (40.0%)
Number of nodules in each patient	1	26 (86.7%)	11 (84.6%)	15 (88.2%)
	2	2 (6.7%)	1 (7.7%)	1 (5.9%)
	3	2 (6.7%)	1 (7.7%)	1 (5.9%)

^†^Locations: Right upper lobe (RUL), Right middle lobe (RML), Right lower lobe (RLL), Left upper lobe (LUL), Left lower lobe (LLL)

### Localization outcomes

The mean time taken for the localization procedure was 46.7 (SD 15.54) minutes in the pre-operative group and 50 (SD 23.59) minutes in the intra-operative group.

The median effective radiation dose in the pre-operative group was 10.4 (IQR 3.9 to 14.7) mSV, including a median of 0.9 mSV additional radiation exposure in the hybrid OR for reconfirmation of hookwire positioning prior to surgery, which was not statistically significant (*p* = 0.87). The median effective radiation dose in the intra-operative group was 3.2 (IQR 1.9 to 4.4) mSV. Using the Mann Whitney test, the pre-operative group had a higher mean rank (262.00) compared to the intra-operative group (203.00) suggesting a higher radiation dose (U = 50.00, *p* = 0.01).

**Fig. 5:**
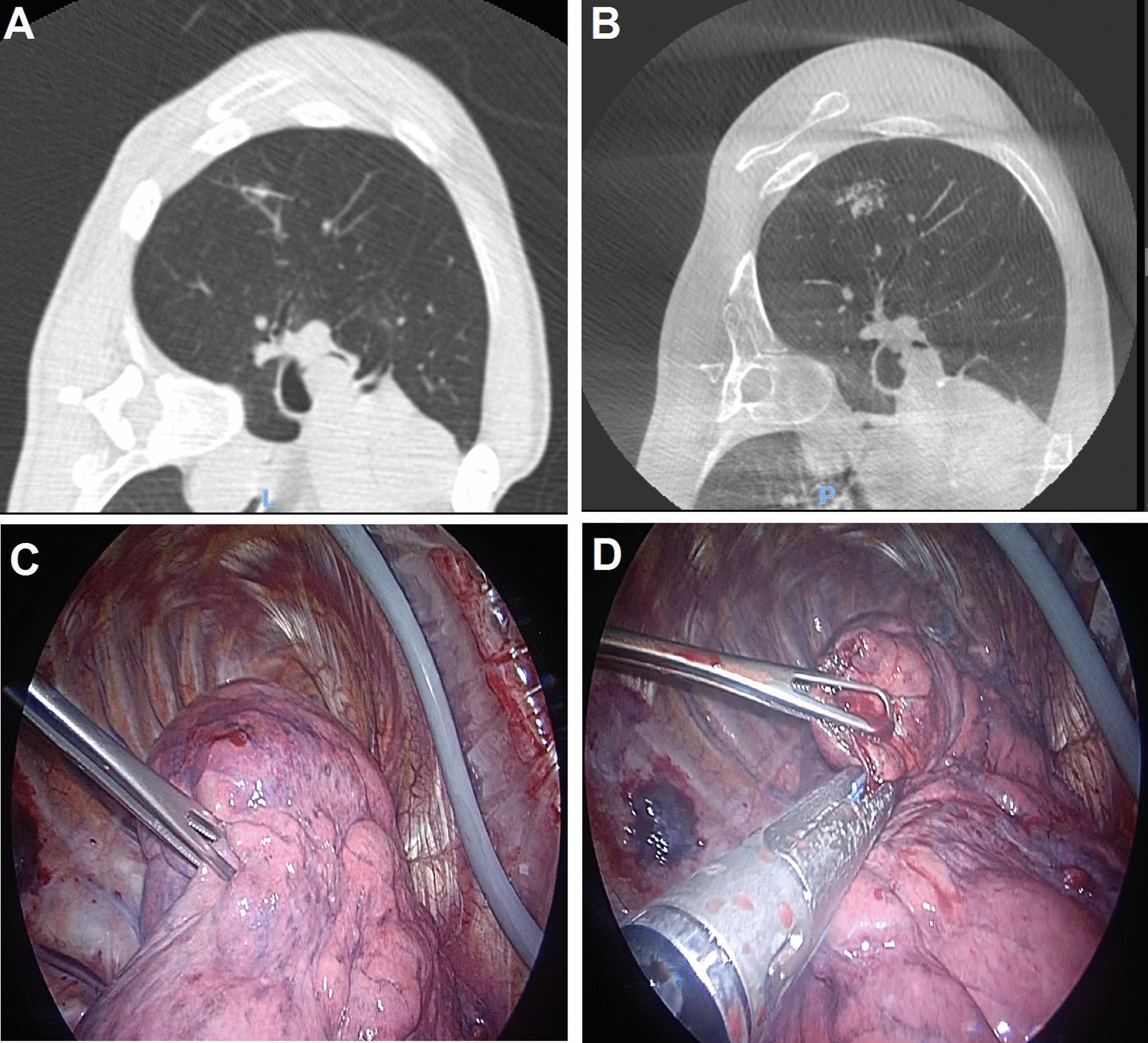
Intra-procedural and intra-operative images depicting nodule localisation and subsequent resection **A** CT scan showing that the hookwire placed in IR suite was within lesion of interest prior to transfer to hybrid OR. **B** Pre-operative cone-beam CT showing complete hookwire dislodgement, but the resulting surface hemorrhage could be used to guide subsequent resection of the lesion of interest. **C** Intra-operative view of the surface hemorrhage seen on pre-operative cone beam CT. **D** Wedge resection of the lesion of interest performed using the previously seen surface hemorrhage as a landmark, and depth of resection was guided by measuring depth of lesion of interest from the surface hemorrhage seen on pre-operative cone-beam CT

There were seven patients (54.0%) in the pre-operative group who had a complication from localization comprising three patients with a minor pneumothorax requiring no intervention, three patients with a significant pneumothorax requiring aspiration and two patients with a hookwire or coil dislodgement. In the intra-operative group, there were also seven patients (41.0%) who had a complication from localization comprising five patients with a minor pneumothorax requiring no intervention, one patient with a significant pneumothorax requiring aspiration and one patient with a dislodgement of the physical marker. Most cases of hookwire or coil dislodgement encountered were partial, with the physical marker still partially lodged in the lung parenchyma to guide surgical resection. However, in one case of pre-operative localization prior to our adjunct use of ICG, there was complete hookwire dislodgement out of the patient’s chest prior to surgery. This nodule was still successfully resected using the pre-operative cone-beam CT to visualize the resultant surface haemorrhage which guided subsequent resection (Fig. 5A–D).

Table 2 summarizes the localization outcomes.

**Table 2:** Localization outcomes

Localization technique used for each nodule			
	Total nodules (n = 36) *n (%)*	Pre-op localization (n = 16) *n (%)*	Intra-op localization (n = 20) *n (%)*
Hookwire only	9 (25.0%)	4 (25.0%)	5 (25.0%)
Hookwire + ICG^†^	21 (58.0%)	8 (50.0%)	13 (65.0%)
Hookwire + ICG^†^ + Lipiodol	3 (8.0%)	1 (6.0%)	2 (10.0%)
Microcoil + ICG^†^	3 (8.0%)	3 (19.0%)	0 (0.0%)

Summary of localization procedural complications			
	Total Patients (n = 30) *n (%)*	Pre-op Localization (n = 13) *n (%)*	Intra-op Localization (n = 17) *n (%)*
All	14 (47.0%)	7 (54.0%)	7 (41.0%)
Minor pneumothorax^‡^	8 (26.7%)	3 (23.1%)	5 (29.4%)
Significant pneumothorax^‡^	3 (10.0%)	2 (15.4%)	1 (5.9%)
Hookwire/ Coil dislodgement	3 (10.0%)	2 (15.4%)	1 (5.9%)

Statistical analyses of outcomes												
Outcome	Mean (SD) or Median (IQR)							Mean Difference (95% CI) or Median Difference		*t*-Statistic (df) or U-statistic		*p*-Value
	Total patients (n = 30)			Pre-op Localization (n = 13)		Intra-op Localization (n = 17)						
Localization duration (min)	48.6 (20.2)			46.7 (15.5)		50.0 (23.6)		− 3.3 ( − 18.8 to 12.2)		− 0.4 (28)		0.66^*^
Effective radiation dose (mSV) ^§^	4.2 (2.3 to 10.5)			10.4 (3.9 to 14.7)		3.2 (1.9 to 4.4)		7.2		50.00		0.01**

Outcome			Total Patients (n = 30) *n (%)*		Pre-op Localization (n = 13) *n (%)*		Intra-op Localization (n = 17) *n (%)*		*χ*^2^—statistic (df)		P-value	
Complication		Yes	14 (47.0%)		7 (54.0%)		7 (41.0%)		0.475 (1)		0.5**	
		No	16 (53.0.%)		6 (46.0%)		10 (59.0%)					

^†^Indocyanine green

^‡^Minor pneumothorax is defined as pneumothorax requiring no intervention while significant pneumothorax is defined as pneumothorax requiring aspiration

*Two-tailed independent samples *t*-test

**Mann Whitney test

^§^The total radiation exposure (mSV) including that in the IR suite as well as confirmatory cone beam CT in the hybrid OR (10.4, IQR 3.9 to 14.7) was not significantly different (p = 0.87) from the radiation exposure in the IR suite alone (9.4, IQR 3.4 to 14.0)

**χ^2^ -square test for independence

Complications arising from localisation included minor pneumothorax requiring no intervention, significant pneumothorax requiring aspiration and physical marker (hookwire or coil) dislodgement

### Surgical outcomes

25 patients underwent VATS wedge resection alone, while five patients underwent VATS wedge resection for the primary lesion of interest requiring nodule localization together with a concomitant lobectomy for a separate lesion in another lobe after the wedge resection. All 36 lung nodules in this series were successfully resected with VATS wedge resection without need for conversion to thoracotomy or a more extensive resection. The mean surgical duration for all resections was 103.2 (SD 43.21) min while the mean intra-operative blood loss for all resections was 31.0 (SD 49.85) ml.

The post-operative course for these patients was generally uneventful with a post-operative hospital stay that ranged from 2 to 13 days (mean 5.43, SD 2.96). There were no intra-operative complications or post-operative mortalities. Post-operative complications included five patients with prolonged air leak, defined as air leak present in the chest tube beyond the 5th post-operative day, (four of which were in patients who had a previous ipsilateral lobectomy), one patient with atrial fibrillation, one patient with pneumonia and one patient with chylothorax (in a patient who underwent concomitant lobectomy and lymph node dissection). There were no requirements for admission to the intensive care unit, intubation or any invasive support. All complications were appropriately treated with medical management and resolved at time of discharge from hospital.

Moreover, there was no statistically significant difference in mean surgical duration, intra-operative blood loss, length of post-operative hospital stay, and frequency of post-operative complications between the two groups.

Table 3 summarizes the surgical outcomes.

**Table 3:** Surgical outcomes

Surgical technique used for each patient			
	Total Patients (n = 30) *n (%)*	Pre-op Localization (n = 13) *n (%)*	Intra-op Localization (n = 17) *n (%)*
Wedge	25 (83.0%)	11 (85.0%)	14 (82.0%)
Wedge + Lobectomy	5 (17.0%)	2 (15.0%)	3 (18.0%)
Conversion to thoracotomy	0	0	0
Successful resection of lesion with wedge resection	30 (100.0%)	13 (100.0%)	17 (100.0%)

Summary of post-surgical complications			
	Total Patients (n = 30) *n (%)*	Pre-op Localization (n = 13) *n (%)*	Intra-op Localization (n = 17) *n (%)*
All	8 (27.0%)	3 (23.0%)	3 (19.0%)
Prolonged air leak	5 (19.0%)	2 (18.0%)	3 (19.0%)
Atrial Fibrillation	1 (4.0%)	0	1 (6.0%)
Pneumonia	1 (4.0%)	0	1 (6.0%)
Chylothorax	1 (4.0%)	1 (9.0%)	0

Statistical analyses of outcomes						
Outcome	Mean (SD)			Mean Difference (95% CI)	*t*-statistic (df)	*p*-Value
	Total Patients (n = 30)	Pre-op Localization (n = 13)	Intra-op Localization (n = 17)			
Surgery duration (min)	103.2 (43.2)	105.0 (46.8)	101.8 (41.7)	3.2 (29.9 to 36.4)	0.2 (28)	0.84*
Intraoperative blood loss (ml)	31.0 (49.9)	33.1 (42.5)	29.4 (56.1)	3.7 ( − 34.6 to 41.9)	0.19 (28)	0.84*
Post-operative hospital stay (days)	5.4 (3.0)	5.0 (2.7)	5.7 (3.2)	− 0.8 ( − 3.0 to 1.5)	− 0.69 (28)	0.49*
Mortality	0	0	0			

Outcome		Total patients (n = 30) *n (%)*	Pre-op Localization (n = 13) *n (%)*	Intra-op Localization (n = 17) *n (%)*	*P*-Value
Complication	Yes	8 (27.0%)	3 (23.0%)	5 (29.0%)	> 0.99**
	No	22 (73.0%)	10 (77.0%)	12 (71.0%)	

*Two-tailed independent samples *t*-test

**Fisher’s exact test

Post-operative complications included prolonged air leak (n = 5), atrial fibrillation (n = 1), pneumonia (n = 1) and chylothorax (n = 1)

Final histology showed three benign (7%) and 33 malignant (93%) nodules, including both primary lung cancer or pre-malignant lesions (n = 31) as well as metastatic lesions (n = 2) (Table 4). All mixed or ground glass nodules resected demonstrated a primary lung cancer or pre-malignant lesion on histopathology. In contrast, only 62% of solid nodules resected demonstrated a malignant lesion, while the remaining 38% nodules demonstrated a benign lesion. This was statistically significant with a P-value of 0.04. The parenchymal resection margin for malignant nodules (including primary lung cancer, pre-malignant and metastatic lesions) ranged from 3 to 25 mm (mean 9.8, SD 5.43) with no statistically significant difference between the pre and intra-operative groups.

**Table 4:** Lung nodule histopathology

Lung nodule histopathology with correlation to radiological characteristic			
Histology	Radiological Characteristic		
	All Nodules (n = 36) n (%)	Solid (n = 13) n (%)	Mixed or Ground-glass (n = 23) n (%)
Primary Lung Cancer or Pre-Malignant Lesions	31 (86.0%) Invasive Adenocarcinoma: 24 Invasive Mucinous Adenocarcinoma: 2 Minimally Invasive Adenocarcinoma: 3 Atypical Adenomatous Hyperplasia: 2	8 (62.0%)	23 (100.0%)
Metastatic Lesions	2 (7.0%) Leiomyosarcoma: 1 Thymoma: 1	2 (15.0%)	0
Benign Lesions	3 (7.0%) Scar Tissue: 2 Intraparenchymal Lymph Node: 1	3 (23.0%)	0

Statistical analyses of lung nodule histopathology					
Histology		All Nodules (n = 36) n (%)	Solid (n = 13) n (%)	Mixed or Ground-glass (n = 23) n (%)	*p*-Value
Malignant	Yes	33 (92.0%)	10 (77.0%)	23 (100.0%)	0.04^§^
	No	3 (8.0%)	3 (23.0%)	0	

Parenchymal resection margins of primary lung cancer, pre-malignant lesions and metastatic lesions						
	Mean (SD)			Mean Difference (95% CI)	*t*-Statistic (df)	*p*-Value
	Total Nodules (n = 32)^†^	Pre-op Localization (n = 14)^†^	Intra-op Localization (n = 18)			
Parenchymal resection margins (mm)	9.8 (5.43)	9.0 (4.76)	10.4 (5.97)	− 1.39 ( − 5.26 to 2.48)	− 0.73 (30)	0.47

^§^Fisher’s Exact Test

^†^Parenchymal resection data was not available for one pre-malignant nodule in the pre-operative group

Table 4 summarizes the lung nodule pathology.

## Discussion

### Key findings

Our study demonstrates that a multi-disciplinary approach utilizing the hybrid operating room for lung nodule localization allowed for all lesions of interest in both the pre and intra-operative localization groups to be successfully resected with a VATS wedge resection without need for conversion to thoracotomy or a more extensive resection, even in cases where hookwire or coil dislodgement occurred. In addition, no significant complications occurred in either group of patients.

### Strengths and limitations

There are several limitations of our study. First and foremost, as a retrospective, single-institution analysis of a relatively small number of patients, its results may not be generalizable to a broader population. Moreover, we also used a number of localization techniques (hookwire alone, hookwire with ICG and microcoil with ICG) during the 5-year study period which may potentially act as a confounder for our results. However, the outcomes of the study in terms of successful resections and complications during the first two years prior to the use of microcoils or ICG were similar to outcomes during the next three years. In addition, we believe the main reasons for our good outcomes are the proper lesion selection for localization technique (pre-operative or intra-operative), and the use of cone-beam CT to identify the location of the lesion in relation to the physical marker (or surface hemorrhage in the event of complete dislodgement) to guide subsequent resection, which are factors that remained consistent during the entire study period. Finally, this study is predominantly a descriptive analysis of our experience as the selection criteria used to decide the localization technique of choice included specific differences in nodule characteristics, and thus the results in pre-operative and intra-operative localization cannot be directly comparatively analysed.

We believe our study has several strengths that deserve mention. Firstly, all cases in our series had resection performed by a single surgeon, which would remove any confounding due to inter-surgeon variability from surgical technique. We also described a detailed selection criteria to guide selection of hybrid OR assisted pre-operative versus intra-operative localization, and a novel workflow where the hybrid OR cone-beam CT is utilized to re-evaluate patients who have undergone pre-operative localization for patients unsuitable for intra-operative localization to guide subsequent resection even in event of physical marker dislodgement. Moreover, we also demonstrate that the additional radiation exposure from this step is modest compared to overall radiation exposure experienced by patients undergoing pre-operative localization in the IR suite. Our work demonstrates that this approach is safe and highly efficacious, but future larger, prospective and multi-centre studies are required to externally validate our findings.

### Comparison with similar researches

Several localization techniques based on CT guidance have been previously described, including the use of metallic devices (hookwires [9], coils [22]), contrast agents (Lipiodol [23]) or dyes (methylene blue [24] and ICG [25]). In addition, electromagnetic navigation bronchoscopy may also be used to insert mircocoils or inject dyes for lung nodule localization [26]. While several comparative studies on lung nodule localization techniques have been performed [26–28] there is no single modality which has been established to be clearly superior to the others. In our centre, CT guided hookwires were used as the main stay of localization, with contrast and dyes used as adjuncts to increase visibility or as a precaution to allow localization of the nodule intra-operatively even if dislodgement occurred.

The use of a physical device in conjunction with a staining measure allows tenting of the lung parenchymal on VATS-assisted resection, providing an additional helping hand to the surgeon during the surgery. Compared to other methods, the use of hookwires has been described to be more prone to complications related to dislodgement [22, 29]. In our case series, three patients (10%) experienced this complication, with one patient having a complete hookwire dislodgement with subsequent localized haemorrhage at the localization site. The rate of complications could also be related to different level of expertise and experience of the operator, as well as logistical factors such as time elapsed between localization and resection as well as distance and mode of transport of patient in between the procedures. Although the risk of systemic air embolization is low and we did not encounter this within our case series, this remains an important complication to consider given its severity. Care should be taken to review for intravascular air on post-localization CT images (for both intra and pre-operative localization, where positive pressure ventilation in intra-op localization is an additional risk factor), and to assess the patient for any symptoms of neurologic or cardiac ischaemia (for pre-operative localization) both after localization and after patient transfer or repositioning as these are also risk factors for air embolization [30–32]. On the other hand, the coil method often requires intra-operative fluoroscopy for visualisation (especially for deeper lesions). This requires additional lead shielding for the operating team and intrusion of the fluoroscopy unit into the operating field.

In particular, the use of ICG is effective as the use of an ICG compatible camera system allows visualisation of the ICG dye up to a depth of 2 cm [33], allowing the dye to serve as a backup localisation marker in case the needle is dislodged.

### Explanation of findings

The 100% successful resection rate with no conversion from VATS to open surgery or requirement for more extensive resections to remove the lesion of interest can be attributed to the following two factors. Firstly, our multi-disciplinary collaboration between thoracic surgery and interventional radiology with regard to patient selection and pre-procedural planning, including choice of localization technique and planned needle trajectory as described earlier. Secondly, the use of cone-beam CT to identify the location of the lesion in relation to the physical marker (or surface hemorrhage in event of complete dislodgement) to guide subsequent resection.

All cases of pre-operative localization in our case series had a cone-beam CT performed in the hybrid OR just prior to surgical resection. This is unique to our case series. This additional step allows the surgeon to have a precise guide of the location of the lesion in relation to the physical marker (or surface haemorrhage, in event of complete hookwire dislodgement) that allows for accurate resection without requiring conversion to open surgery or more extensive resection. While there is a minor increase in radiation dose to the patient of a median of 0.9 mSV, this is not significant when comparing overall radiation dose of the localization procedure. To our knowledge, we are the only centre routinely using cone-beam CT to reconfirm localization in pre-operative localization cases and this is a major contributing factor to our high success rates not requiring conversion to open surgery or more extensive resections in this group.

### Implications and actions needed

Our experience further confirms that hybrid OR intra-operative localization can be safely performed with a high level of accuracy, regardless of the marker used. We also demonstrate that intra-operative localization results in a comparatively lower radiation dose compared to pre-operative localization, which is similar to the experience in other centres [17, 18]. In addition, our work also shows that the hybrid OR may have a role in improving the accuracy of pre-operative localization done in the IR suite, allowing for re-evaluation with cone-beam CT to guide subsequent resection even in the event of physical marker dislodgement, at only a fairly small additional dose of radiation exposure. The hybrid OR is not a resource which is available to every thoracic surgical unit, so our approach may not be adoptable by many centres. However, as hybrid ORs are becoming increasingly more common [34], we hope our experience will encourage other thoracic surgical units to incorporate the hybrid OR in their practice when the opportunity becomes available to them.

## Conclusions

In our experience, hybrid OR cone-beam CT assisted pre-operative and intra-operative localization are safe and effective measures to facilitate intentional VATS wedge resections of small, deep or predominantly ground-glass lesions. Intra-operative localization is associated with lower radiation doses compared to pre-operative localization, so it may be the favoured approach whenever feasible. However, multidisciplinary collaboration is key in deciding on the best localization technique for each lesion to maximize successful chance of resection. In addition, the routine use of cone-beam CT to confirm the position of the physical marker in relation to the nodule prior to surgery in the hybrid OR helps mitigate consequences of localization failure in patients who undergo pre-operative localization at only a modest increase in radiation exposure.

## Data Availability

The datasets generated and/or analysed during the current study are not publicly available due to strictly controlled access to patient related information by local laws due to privacy concerns.

## Abbreviations

CT: Computed tomography
VATS: Video-assisted thoracoscopic surgery
OR: Operating room
IR: Interventional radiology
ICG: Indocyanine green
DLP: Dose length product
DAP: Dose area product
mSV: MilliSieverts
